# Active post-marketing surveillance of the intralesional administration of human recombinant epidermal growth factor in diabetic foot ulcers

**DOI:** 10.1186/2050-6511-14-44

**Published:** 2013-09-03

**Authors:** Isis B Yera-Alos, Liuba Alonso-Carbonell, Carmen M Valenzuela-Silva, Angela D Tuero-Iglesias, Martha Moreira-Martínez, Ivonne Marrero-Rodríguez, Ernesto López-Mola, Pedro A López-Saura

**Affiliations:** 1 Center for the Development of Pharmacoepidemiology, Havana, Cuba; 2 Center for Genetic Engineering and Biotechnology, Havana, Cuba; 3 “Abel Santamaría” Hospital, Pinar del Río, Cuba; 4 “Gustavo Aldereguía” Hospital, Cienfuegos, Cuba

**Keywords:** Diabetic foot ulcer, Epidermal growth factor, Pharmacoepidemiology

## Abstract

**Background:**

After several exploratory and confirmatory clinical trials, the intralesional administration of human recombinant epidermal growth factor (hrEGF) has been approved for the treatment of advanced diabetic foot ulcers (DFU). The aim of this work was to evaluate the effectiveness and safety of this procedure in medical practice.

**Methods:**

A prospective, post-marketing active pharmacosurveillance was conducted in 41 hospitals and 19 primary care polyclinics. Patients with DFU received hrEGF, 25 or 75 μg, intralesionally 3 times per week until complete granulation of the ulcer or 8 weeks maximum, adjuvant to standard wound care. Outcomes measured were complete granulation, amputations, and adverse events (AE) during treatment; complete lesion re-epithelization and relapses in follow-up (median: 1.2; maximum 4.2 years).

**Results:**

The study included 1788 patients with 1835 DFU (81% Wagner’s grades 3 or 4; 43% ischemic) treated from May 2007 to April 2010. Complete granulation was observed in 76% of the ulcers in 5 weeks (median). Ulcer non-ischemic etiology (OR: 3.6; 95% CI: 2.8-4.7) and age (1.02; 1.01-1.03, for each younger year) were the main variables with influence on this outcome. During treatment, 220 (12%) amputations (171 major) were required in 214 patients, mostly in ischemic or Wagner’s grade 3 to 5 ulcers. Re-epithelization was documented in 61% of the 1659 followed-up cases; 5% relapsed per year. AE (4171) were reported in 47% of the subjects. Mild or moderate local pain and burning sensation, shivering and chills, were 87% of the events. Serious events, not related to treatment, occurred in 1.7% of the patients.

**Conclusions:**

The favorable benefit/risk balance, confirms the beneficial clinical profile of intralesional hrEGF in the treatment of DFUs.

## Background

Pharmacovigilance and post-marketing studies are key elements to monitor the safety and effectiveness of approved drugs. They are excellent scenarios to confirm and extend the safety profile and efficacy data acquired in previous clinical trials.

Products for the treatment of diabetic foot ulcers (DFU) available in the market have been scarcely effective in advanced and/or ischemic lesions [1]. Necrotic tissue, sepsis, inflammation and wound proteases impair the effective distribution of the active pharmaceutical ingredients when they are administered in topically [2].

A new procedure has been recently developed for the treatment of these advanced DFU based on the intralesional infiltration of recombinant, human epidermal growth factor (rhEGF) as a lyophilized formulation under the brand name Heberprot-P®. The rationale of this procedure has been published and reviewed recently [2,3].

Exploratory [4-6] and confirmatory [7] clinical trials have been successfully completed with this product in patients suffering from advanced DFUs with potential amputation risk (reviewed in ref. 3). These trials demonstrated that the intralesional administration of rhEGF accelerates the wound healing process. The generation of useful granulation tissue on the ulcer bed facilitated its closure by either second intention or a skin graft. The product’s safety profile was acceptable and the benefit-risk analysis of its use yielded a largely favorable balance. These results granted the approval by the Cuban Regulatory Authority (CECMED).

In April 2007 this treatment was included in the country’s Basic Drugs List and its use was extended to all Angiology services, firstly in the secondary healthcare level (hospitals) and subsequently in the primary level (polyclinics).

At the same time, postmarketing active surveillance was initiated in order to evaluate the effectiveness and safety of the drug in the current medical practice. This article reports the first results of this surveillance.

## Methods

### Design and participants

A multicenter, prospective, intensive, post-marketing surveillance was conducted on patients treated from May 2007 to April 2010; follow-up extended to December 2011. The study was coordinated by the Center for the Development of Pharmacoepidemiology (CDF) from the Cuban Ministry of Health and its national network. There were 41 participating hospitals from the 15 Cuban provinces and 19 polyclinics from 7 provinces.

Patients, more than 18 years-old, suffering from 1 cm^2^ or larger DFUs were prescribed with rhEGF and included. The indication comprised Wagner’s [8] grades 3 and 4 DFU, but some patients with grades 1, 2, and 5 were included, under off-label use according to the physicians’ decision. Patients with diabetic coma, uncontrolled heart, renal or liver disease, history or suspicion of malignancy, pregnancy or breastfeeding were not treated. Prescription was part of the patient’s regular care, not induced by the present work. The protocol was approved by the CDF Institutional Review Board. The confidentiality of the patients’ personal data was preserved.

Lesion etiology was classified as ischemic or not. When the ankle/brachial index (ABI) was available, patients with values below 0.75 were considered ischemic, following the same criterion used in the clinical trials. Otherwise, clinical signs of ischemia such as absence of pulses (femoral-popliteal, tibial or distal), intermittent claudication, pain, local atrophy, coldness, and hair loss were taken into account for this classification.

Since it was not feasible to standardize the ulcer size measurement among so many clinical sites, lesion extension-location was considered in three categories: (i) simple, if the ulcer covered only one region of the foot (toes, dorsum, sole, internal edge, external edge, except calcaneus); (ii) complex, if the extension of the ulcer comprised several regions but not the heel, and (iii) calcaneal if this region was involved.

### Intervention

The treatment was as in-patients, although ambulatory care was allowed if the subject could attend the treatment visits. The standard care included the patient’s metabolic control, lesion area sanitation and systematic cures, sharp debridement of the necrotic or infected tissue with minor amputation of the affected zones if necessary, and moist gauze dressing. Wide spectrum antimicrobial drugs were prescribed in patients exhibiting local clinical signs of infection. Pressure off-loading of the affected zones was recommended as well.

The product (Heberprot-P®, Heber Biotec, Havana) is lyophilized, containing 75 or 25 μg of rhEGF per vial, to be dissolved with 5 ml of water for injection. In every visit this volume was distributed throughout the lesion in 0.5–1 ml injections. The solution was injected first into the dermo-epidermal junction at equidistant points all over the lesion contours and then downward into the wound bottom to ensure a uniform distribution. The needle was changed for each puncture. The product label indicates injections 3 times per week (tpw) on alternate days. However, in some cases the physicians decided to modify the schedule to daily, twice or once per week. A treatment cycle continued until complete granulation was achieved, lesion closed by autografting or 8 weeks maximum.

The rhEGF dose (25 μg or 75 μg) was selected according to the label (25 μg in ulcers smaller than 20 cm^2^ and non-ischemic), but the choice was also determined by each physician’s criteria, his/her experience with this product, and the availability of either drug presentation in the healthcare unit at a certain moment.

### Outcomes and measurement methods

The main effectiveness variable was lesion complete granulation, evaluated by direct visual inspection. It was defined as productive material, able to mediate the complete lesion closure by second intention or an autologous skin graft. Macroscopically, it was characterized by the presence of reddish, diffused, dispersed and lustrous miliary granular formations that bleed easily after manipulation.

Secondary outcomes were time-to-complete granulation, need for amputation and its type during treatment. During the follow-up period wound closure (complete re-epithelization), relapses, and patient’s survival were evaluated.

The variables used to evaluate safety were the adverse events (AE) during the treatment period, considering the type of event, organ and system affected [9], its seriousness and causality relation with the treatment. The latter was done according to the World Health Organization (WHO) algorithm [10] only for serious adverse events (SAE) by the provincial pharmaco-epidemiologists and a consulting multidisciplinary team created *ad hoc* for this purpose. SAE reported were included in the Cuban Pharmacosurveillance System Database, which guarantees the absence of duplicated data when processing reports received from different sources.

Information was gathered by the physicians in case report forms (CRF) which covered the in-patient treatment period. Patients’ final outcome was collected in a further follow-up visit, done by the hospital or municipality pharmaco-epidemiology staff, scheduled annually. Emphasis was placed on the training of the personnel in all aspects related to the drug safety evaluation.

Deaths during treatment were further investigated by review of the clinical records and, if necropsy was performed, its report. Causes of death were taken from the death certificates, coded according to the International Classification of Disease-10.

The occurrence of any type of cancer was actively investigated through: (i) direct interview to the patient in the follow-up visits; (ii) cross-search in the National Cancer Registry (NCR), or (iii) findings in the National Mortality Registry. The latter cross search was also useful for the identification and confirmation of follow-up period deceases.

### Statistical analyses

One patient could be treated more than once for the same or a different lesion at different moments. Then the experimental unit considered was the treatment cycle, since each of them generated one CRF. Short term AE were evaluated on this basis. The lesion was the unit taken into account for the analyses of granulation, amputations, healing, and relapses. Survival and long term AE were done on patient basis.

The statistical treatment of the data was conducted with the PASW 18.0 software. Measures of central tendency and dispersion such as mean, median, standard deviation (SD), 95% CI, quartile range (QR), minimal and maximal values, were calculated to describe quantitative variables. Graphical normality analysis (QQPlot) and Kolmogorov-Smirnov test for goodness of fit were applied. The chi-square values were calculated to evaluate dependency among qualitative variables. Time variables were estimated using Kaplan-Meier plots and compared with the log-rank test. Logistic or Cox regression models were adjusted to assess the influence of control variables on the outcomes. For the variable-association analyses only the results from the first treatment cycle on each patient were taken into account to avoid considering non-independent observations. Safety related variables were subjected to descriptive statistics. A Bayesian approach was used for the benefit-risk ratio analysis.

## Results

### Characteristics of the subjects

CRFs were available from 1788 subjects bearing 1835 DFU. This population represents approximately 80% of all the DFU patients treated with rhEGF in the study period, according to weekly reports received at CDF. Of them, 1676 (93.7%), were seen in hospitals and 112 (6.3%) in polyclinics. Most of the patients (1729; 96.7%) received one treatment cycle; 56 (3.1%) were given two cycles; four of them simultaneously on two different lesions and 3 (0.2%) had three cycles. Therefore the whole patient population received 1851 treatment cycles with rhEGF.

The main demographic characteristics are shown in Table 1. Females, white colored, and diabetes type 2 were predominant. Patients older than 75 years were 248 (14%). The main co-morbidities were hypertension, ischemic cardiopathy, previous DFU, and amputations. Ulcers were mostly advanced (82% Wagner’s grades 3–5). Ischemic lesions were 34% diagnosed clinically and 9% by ABI. Simple lesions (mainly on the toes) were the most frequent.

**Table 1:** Clinical and demographical characteristics of the patients or ulcers

**Characteristics**		**Results (N = 1788 patients)**
Age (years): median ± QR (minimum; maximum)		65.0 ± 14.0 (19; 98)
Gender: masculine/feminine (% feminine)		825/963 (53.9%)
Ethnic groups (24 missing)	White	1132 (63.3%)
	Black	289 (16.2%)
	Mixed	333 (18.6%)
	Chinese	10 (0.6%)
Diabetes mellitus: type 1/type 2 (% type 2) (47 missing)		379/1362 (76.2%)
Smokers (6 missing)		368 (20.6%)
Arterial hypertension (4 missing)		1085 (60.7%)
Ischemic cardiopathy (9 missing)		411 (23.0%)
Antecedents of foot ulcers		662 (37.0%)
Antecedents of amputations		420 (23.5%)
		**N = 1835 ulcers**
Etiology of the ulcer (1 missing)	Ischemic	790 (43.1%)
	Non ischemic	1044 (56.9%)
Location-extension of the lesion (118 missing)	Simple	1140 (62.1%)
	Complex	354 (19.3%)
	Calcaneal	223 (12.2%)
Wagner’s classification (83 missing)		
	Grade 1	26 (1.4%)
	Grade 2	228 (12.4%)
	Grade 3	981 (53.5%)
	Grade 4	504 (27.5%)
	Grade 5	13 (0.7%)

### Treatment compliance

There were some treatment schedule deviations according to doctors’ decisions: 267 Wagner’s grade 1, 2, or 5 ulcers (15% of all lesions) were treated as off-label indications; 281 ischemic lesions (36%) were treated with the 25 μg dose; 50 lesions were treated with both doses, considered as 75 μg for the analyses; the three tpw schedule was not followed strictly (see next paragraph); 48 treatment cycles comprised more than 24 injections.

The 75 μg dose was given more frequently to Wagner’s grades 3–5 ulcers (61%) than to less severe cases (53%) and to patients with ischemia (64%) than without it (54%). The thrice weekly regime was the most frequently used. However, some cases were treated daily (9%) or less than three tpw (14%). The median number of infiltrations was 10 (range 1–47). The median total exposure to rhEGF was 500 μg (range 25–3825).

Interruptions occurred in 462 treatment cycles (25%). The causes were: voluntary abandon in 200 (10.8%) cases; worsening of the lesion in 207 (11.2%); local hypergranulation in 16 (0.9%), and other AE in 39 (2.1%).

### Granulation

Complete granulation was achieved in 76% of the ulcers at the end of treatment (Table 2). This favorable response was more likely to occur in patients without clinical manifestations of ischemia. The other variables with significant enhancing influence on the granulation outcome in bivariate analyses were age ≤ 75 years better than older (78% vs. 59%), non-smoker (77% vs. 68%), three tpw schedule better than <3 tpw (78% vs. 65%), non-calcaneus location (77% vs. 66%), non-history of hypertension (79% vs. 73%), non-history of ischemic cardiopathy (78% vs. 66%), and the 25 μg dose (81% vs. 72%). In a multivariate, logistic regression model granulation response was favored by non-ischemia (OR: 3.6; 95% CI: 2.8-4.7), non-smoking (1.3; 1.01–1.8), age (1.02; 1.01–1.03, for each younger year), three tpw schedule (1.9; 1.4–2.7), and the 25 μg dose (1.6; 1.2–2.0). The healthcare level, gender, ethnic group, and Wagner’s classification were not significantly related to the granulation outcome. The time-to-complete granulation differed significantly between non-ischemic and ischemic lesions (log-rank test; p < 0.001).

**Table 2:** Lesion response to treatment according to pathogeny

		**Ischemic**	**Non-ischemic**	**Total**
Complete granulation		486/790	905/1044	1392/1835
	%	61.5	86.7	75.9
	(95% CI)	(58.1; 64.9)	(84.6; 88.7)	(73.9; 77.8)
Weeks to complete granulation median (95% CI)		6 (5.6; 6.3)	4 (3.8; 4.2)	5 (4.8; 5.2)
Healing		371/742	641/916	1012/1659
	Evaluated (“per protocol”) %	50.0	69.9	61.0
	(95% CI)	(48.1; 54.9)	(66.9; 73.3)	(58.2; 62.9)
	Included (“intention-to-treat”) %	47.0	61.4	55.1
	(95% CI)	(43.4; 50.4)	(58.4; 64.4)	(52.9; 57.4)
Relapses: (years-person of follow- up)		26/333 (561)	49/584 (927)	75/917 (1488)
	Rate per year (95% CI)	4.6 (2.9; 6.4)	5.3 (3.8; 6.7)	5.0 (3.9; 6.2)
Amputations during treatment		180/790	40/1044	220/1835
	%	22.8	3.8	12.0
	(95% CI)	(19.9; 25.7)	(2.7; 5.0)	(10.5; 13.5)

### Amputations

At the end of treatment 214 patients required 220 amputations (12%) (Table 2); 23 of them were disarticulations, 26 transmetatarsal and 171 major amputations (9.3% of the lesions). Ischemic ulcers caused more amputations. Most of the amputations (85%) were in cases with Wagner’s 3–5 ulcers. Calcaneus location of the ulcers was also an unfavorable factor for the amputation outcome (18.5% vs. 11.0% in non-calcaneus ulcers). Smoking habit (17% vs. 11% in non-smokers) and history of previous amputation (15% vs. 11%) conditioned a higher amputation rate too.

### Adverse events

A total of 4171 AE were reported (70 different types) in 46% (856/1851) of the treatment cycles in 838 subjects (47%). The AE occurring in more than 1% of the patients and the maximal number of repetitions of the event in any subject are summarized in Table 3. Pain and burning sensation at the administration site, shivering and chills account for 87% of all the AE reported. Their frequency decreased as the treatment continued: from more than 14% in the first application to less than 2% after 40 days. More than 85% of the events were mild or moderate and easily manageable. Except for local infection, all common AE were more frequent with the 75 μg dose. The rate of local infection as an AE (4%) was not dependent on the baseline infection status of the ulcer (Table 4).

**Table 3:** Frequent adverse events (> 1.0%)

**Type of event**	**Treatment cycles N = 1851**		**Maximum number of repetitions**
	**N**	**%**	
Pain at the administration site	402	21.7	24
Shivering	368	19.9	23
Burning sensation at the administration site	297	16.0	17
Chills	171	9.2	16
Local infection	70	3.8	4
Fever	42	2.3	7
Ulcer worsening*	30	1.6	2
Vomits	25	1.4	10

*:Reported by the doctors as adverse events. In fact, worsening of the ulcer as a therapeutic failure occurred in 209 cycles (11.3%).

**Table 4:** Local infection as an adverse event vs. baseline

		**Occurrence of local infection as adverse event**		**Total**
		**Yes**	**No**	
Local infection as baseline	Yes	52	1270	1322
	No	15	358	373
Total		67	1628	1695

Information on baseline infection was missing in one subject with local infection as adverse event and in 90 subjects without it. The OR was 0.98; 95% CI: 0.54–1.76.

The 31 SAE reported are shown in Table 5. Two of them (ketoacidosis and gastroenterocolitis) were unrelated to the treatment. The other 29 were classified as conditional or possible, since there was a temporal relation with treatment but there are alternative explanations to the clinical findings. Local infection accounted for 32% of the SAE and cardiovascular syndromes for 14 SAE. Thirteen of the latter patients had antecedent of cardiovascular disease (hypertension, ischemic cardiopathy, or arrhythmia).

**Table 5:** Serious adverse events

**Motive for seriousness**	**Events**	**N**	%*
Caused death	Acute myocardial infarct	5	
	Sudden cardiac death	2	
	Acute pulmonary edema	2	
	Ventricular fibrillation	1	
	Uncompensated heart insufficiency	1	
	Ischemic stroke	1	
	Acute respiratory failure	1	
	Acute gastroenterocolitis	1	
	Diabetic ketoacidosis-septic shock	1	
	Subtotal	15	0.8%
Endangered life	Acute pulmonary edema	2	
	Loss of consciousness	2	
	Local infection	1	
	Glottic spasm	1	
	Faintness	1	
	Subtotal	7	0.4%
Prolonged hospitalization	Local infection	4	0.2%
Produced disability	Local infection	5	0.3%
TOTAL		31	1.7%

*Percent calculated from 1851 treatment cycles.

**Figure 1:**
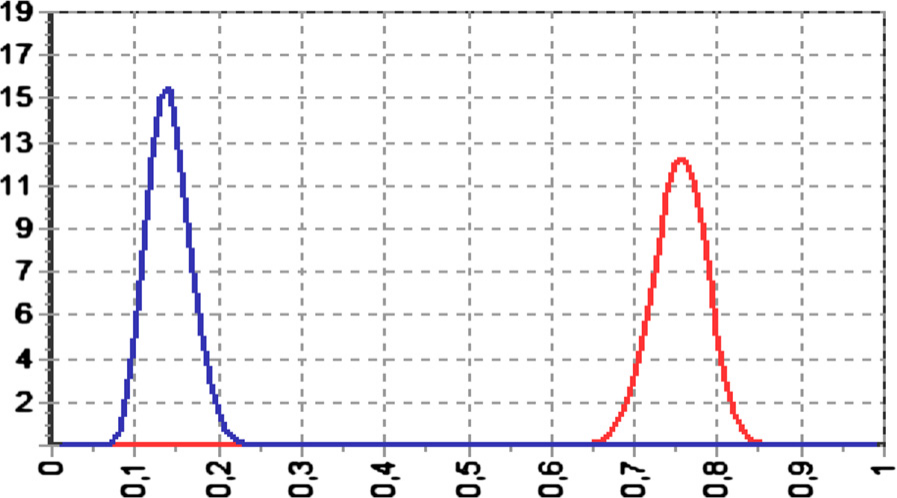
**Risk-benefit analysis.** Given *p*(*x | benefit*) by the probability distribution function for benefit (complete granulation) and *p*(*x* | *risk*) by the probability distribution function for risk (moderate and severe adverse events or amputation) then the Bayes Factor (B_br_) is: $B_{\mathrm{br}}=\frac{p\left(x\;|\;\mathrm{benefit}\right)}{p\left(x\;|\;\mathrm{risk}\right)}$ representing a summary of the evidence provided by the data in favor of benefit (red), as opposed to risk (blue). A value larger than 1 means a favorable benefit-risk ratio. In this case: Bayes factor = 5.4; difference between probabilities: 61% (95% CI: 59%–64%).

The benefit/risk ratio is presented in Figure 1. The odds for benefit were larger than for risk (Bayes Factor = 5.4; difference between probabilities: 61%; 95% CI: 59%–64%).

### Follow-up: wound closure

Post-treatment follow-up information was obtained from 1620 subjects (91%) with 1659 ulcers, at least once during the 4 years after the end of treatment. The follow-up period (median: 1.2; maximum 4.2 years) comprised 2270.6 years-person. The baseline and demographic characteristics of the followed-up subjects do not differ from the whole patient population (data not shown). Complete ulcer re-epithelization was achieved in 55% of all the lesions (“intention-to-treat” evaluation), 61% of those evaluated (Table 2). In a logistic regression model significant positive influence on this outcome was shown by: non-ischemic etiology (OR: 2.1; 95% CI: 1.6–2.6), age (1.012; 1.002–1.022, for each younger year), and not-history of amputation before treatment (1.7; 1.3–2.1). Relapses occurred in 5% person-years, regardless of the ulcer etiology or other characteristics (Table 2).

### Survival and long-term safety

**Figure 2:**
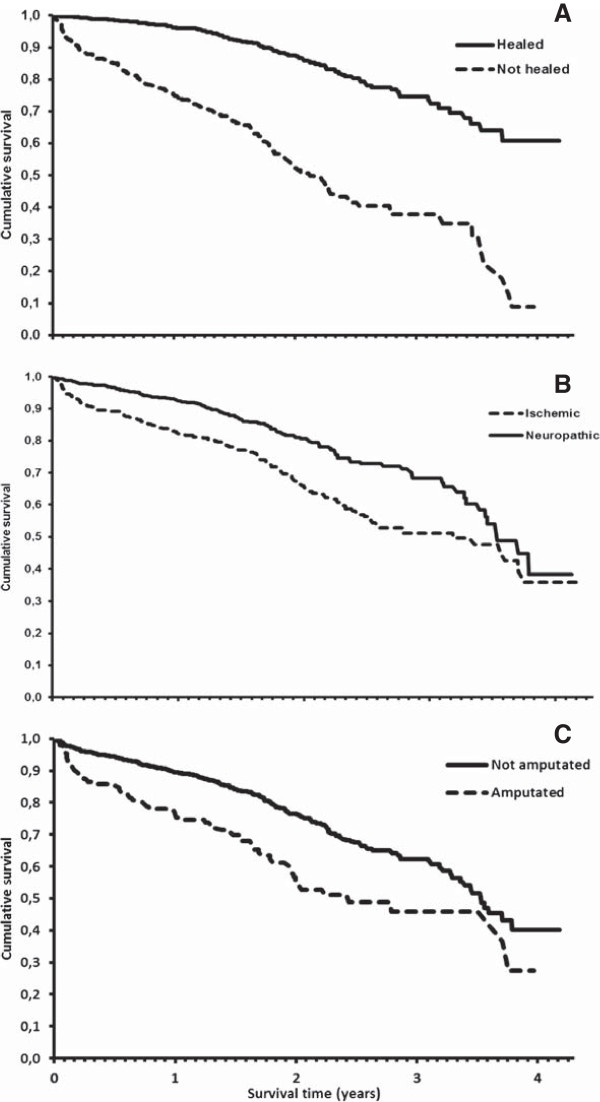
Kaplan-Meier curves for survival time according to A) whether the ulcer healed or not; B) ulcer etiology or C) whether the affected limb was amputated or not.

During treatment or follow-up 352 patients (20%) died. Table 6 lists the causes of death. The most frequent were cardiovascular disorders (41.8%); among them acute myocardial infarct and ischemic cardiopathy. Diabetes itself and its complications (renal disease and others) represented 17%, and tumors caused 8% of the deceases. A Cox-regression yielded that the variables with significant unfavorable influence on survival were (HR; 95% CI): ischemic etiology (1.4; 1.1–1.8), history of ischemic cardiopathy (1.3; 1.01–1.6), and older age (1.03; 1.01–1.04 per year increment), whereas ulcer healing showed a significant protective effect (0.25; 0.19–0.31). Amputation after treatment had an inverse lineal correlation with healing. Figure 2 illustrates the effects of healing, etiology, and amputation on survival through the corresponding Kaplan-Meier plots.

**Table 6:** Causes of death in diabetic foot ulcer patients treated with intralesional hrEGF

**Cause**	**Number**	**Percent of all deaths**	**Rate (per 100 person-years)**
Cardiovascular disorders	145	41.2%	6.38%
Acute myocardial infarct	37	10.5%	1.63%
Stroke and its sequels	33	9.4%	1.45%
Ischemic cardiopathy	29	8.2%	1.28%
Cardiac failure	18	5.1%	0.79%
Hypertensive cardiac disease	9	2.6%	0.40%
Other: peripheral angiopathy (5); acute pulmonary edema (4); cardiac arrhythmias (4); cardiac arrest and cardiogenic shock (3); generalized atherosclerosis; non specified (2); abdominal aorta aneurism rupture (1)	19	5.4%	0.84%
Other complications of diabetes	60	17.0%	2.64%
Diabetic coma	6	1.7%	0.26%
Diabetes mellitus with renal complications	30	8.5%	1.32%
Diabetes mellitus with other complications	13	3.7%	0.57%
Diabetic foot	11	3.1%	0.48%
Respiratory disorders	53	15.1%	2.33%
Pneumonia and bronchopneumonia	49	13.9%	2.16%
Acute respiratory insufficiency	4	1.1%	0.18%
Neoplasia: breast (8); prostate (3); colon (4); bladder (2); lung (3); peritoneum (2); pancreas (1); kidney (1); pharynx (1); spinal column (1); mouth (1); tumor of unknown behavior, encephalus, suptratentorial (1)	28	8.0%	1.23%
Peripheral venous thrombosis and thromboembolism	12	3.4%	0.53%
Amputations and other surgical complications	12	3.4%	0.53%
Other causes: sepsis (8); upper digestive bleeding (4); gastroenteritis (2); liver cirrhosis (2); rheumatoid arthritis (1); viral encephalitis (not specified) (1); multiorgan failure (1); alcoholic hepatitis (1); prostate hyperplasia (1); accidents and traumatisms (2); sudden death (1)	24	6.8%	1.06%
Unknown	18	5.1%	
Total	352		15.50%

(From the national death certificate database).

During the follow-up 42 subjects were identified with neoplasia, not diagnosed before treatment. None of them was on the rhEGF treated region. Locations were: breast (11), colon (5), prostate (4), uterus (4), bladder (2), lung (3), peritoneum (2), and skin basal cell, rectum, endometrium, stomach, fibrosarcoma, pancreas, kidney, pharynx, spinal column, mouth, and not specified, one each. Cancer was not related to the extent of exposure to rhEGF treatment: all these patients had received one treatment cycles; the average exposure was 535 μg of rhEGF (range: 75–2100 μg).

## Discussion

The postmarketing surveillance covered all the Angiology wards that manage DFU in Cuba and some primary care units that have incorporated this drug as part of the diabetic patients integral care program. The demographic and baseline characteristics of the subjects parallel those of people with DFU that took part in the clinical trials with this procedure [3-7]: predominantly diabetes type 2, median age 65 years, approximately equal gender distribution, high proportions of hypertension, ischemic cardiopathy, and previous ulcers, as well as the ethnic distribution of the Cuban population [11]. Co-morbidities such as hypercholesterolemia, arterial hypertension and ischemic cardiopathy have been reported in other settings [12] too. Nearly half of the subjects had ischemic ulcers, mostly Wagner’s grades 3–4, which singularizes the application of intralesional hrEGF from other procedures that are mainly indicated in less advanced lesions [13]. Calcaneus ulcers were not excluded, which is also an unfavorable condition [14].

Some deviations from the labeled pattern of hrEGF application were seen, which were due to doctors’ preference, according to their judgment of the patient’s evolution. In some cases, ambulatory patients could not attend all the visits. The dose deviations were also determined, eventually, by product availability at a given moment. The off-label use of medicaments, particularly biologics, is not a seldom practice in many settings as part of the common medical practice [15,16].

Treatment interruptions were mainly due to failure that determined amputation mostly in ischemic, Wagner’s 3–5 lesions. Very few were because of patients’ intolerance (AE or voluntary abandonments). Hypergranulation is noteworthy since it is due to the same mechanism of healing enhancement. It was easily controlled by tissue excess removal and there is no information of related sequels, such as hypertrophic scars or keloids.

The results of the previous clinical trials with intralesional hrEGF (79.4% granulation in 296 patients; 95% CI: 74.8–84.0%) were confirmed in this study. The re-epithelization rate found falls within the pooled analysis of the clinical trials too: 59.2%; 95% CI: 53.4– 64.5% [3]. Granulation response showed good predictive value for final healing in this series [17]. The relapse rate (5% year-persons) is better than reported [18,19]. For example, Boulton et al. [20] refer an estimate of 50–70% for the UK and 34% after one year follow-up in other series. The fact that postmarketing data confirm the results of the clinical trials is an important feature that endorses the use of intralesional rhEGF in medical practice for the treatment of advanced DFU. Topical EGF [21,22] or other growth factors [23,24] have been reported in smaller and/or less advanced ulcers. However, there is scarce information on their effectiveness in regular medical practice or have not fulfilled the expectations raised from clinical trials [25].

The main variables with influence on the treatment effectiveness, both for granulation and re-epithelization, were ischemia and age, which are well-known adverse prognosis factor for ulcer healing [26]. However, the granulation and healing rates attained indicate that the effect of the product is evident in ischemic patients too. Ischemia was considered when ABI < 0.75 or clinically. The ABI cutoff for ischemia varies among different guidelines, from 0.7 [27,28] to 0.9 [29]. Consequently, 40 subjects (85% granulation; 80% re-epithelization) classified as non-ischemic in this work would fall into a “mild” ischemia category (with scarce clinical expose) according to other thresholds. This supports the notion that the product is also effective in patients with impaired blood supply.

Although this work did not include a non-treated control group to measure the impact of the treatment on amputation rate, other independent investigations in additional patient series have made such comparisons. González-Acosta et al. found a major amputation rate reduction from 26.7% to 8.3% when intralesional rhEGF was added to conventional standard care [30]. García-Herrera et al. report a 43.1% to 8.1% major amputation rate reduction [31]. These data are consistent with the 9.2% major amputation rate found in the present work.

The most frequently observed AE such as pain and burning sensation at the injection site, shivering, chills, and local infection coincide with the reported intralesional hrEGF safety profile [3-7].

Local infection is one of the most fearsome AE because it frequently leads to amputation. Approximately 60% of amputations are preceded by infected ulcers [32]. Local infection was classified with “possible” causality relationship with the treatment. This is difficult to evaluate since most of these ulcers were infected at baseline. This diagnosis was defined by signs such as edema, ulcer border redness, and secretion. Heberprot P® labeling says that these signs should be cleared (antibiotics, debridement) before the product is applied. Even though, a negative culture is never obtained. It is unlikely that the injection procedure contributes to spread any subclinical infection as shown by the fact that the occurrence of this AE was not associated to the baseline infection of the ulcer.

There is not enough information to relate cardiovascular AE and deaths to the treatment, since the patients have frequent antecedents of cardiovascular diseases. The relationship of diabetes and cardiovascular diseases is well known, moreover if DFU is present [33,34]. In data from the national death certificate database and the Annual Health Report [35] for 2007–2010, the death rates due to these causes in people with diabetes among the three main causes of death in the certificate double that of the general population. This death profile agrees with the literature for DFU patients [36,37].

A particular concern on the use of growth factors is the possibility of development or stimulation of a pre-existing malignancy. Contrary to PDGF, EGF cannot initiate malignant transformation [38] and results with EGF in experimental models have not demonstrated tumor promotion consistently [3,39]. Diabetes is a known cancer risk boosting condition [40-43]. The data do not support any cancer promotion by rhEGF treatment. Giovannucci et al. discuss the possible mechanisms of the diabetes-cancer association, either by common risk factors or specific mechanisms in diabetes metabolism and management [44]. This aspect has to be further investigated and followed in the product risk-management program.

## Conclusions

In summary this postmarketing, prospective study is consistent with earlier findings from exploratory and confirmatory clinical trials, while extending the data to a larger clinical setting. The positive findings, related to the clinical evolution of the patients treated at secondary and primary Cuban healthcare levels, suggest a relevant role of this drug in the treatment of DFUs. Further clinical research and post-marketing information from other countries should enrich the evidence shown in this paper.

## Appendix

### (Cuban Group for the Introduction of Heberprot P in Diabetic Foot Ulcers)

Patient recruitment, treatment and follow-up.

*Health Unit, Province (number of treatment cycles performed), investigators*: “Camilo Cienfuegos” Hospital, Sancti Spíritus (211): Regla Soto-Águila (province coordinator), Pablo Sánchez Pentón, Irelio Borroto Carpio, Reidel Veloso, Yanet Hernández, Ana Tavio Reyes, Mayilé Gómez, Liliam Moreno Perera; “Gustavo Aldereguía Lima” Hospital, Cienfuegos (154): Ivonne Marrero Rodríguez, Belkis Calaña González Posada, Ivette García, Mabel Medina, Nancy Ramírez, Sahily González Acosta, Yadira Alonso, Javier Borrego; “Abel Santamaría” Hospital, Pinar del Río (128): Martha Moreira Martínez, Aida Hernández, Antonio Díaz Díaz, Agustina Gómez García Yanet Crespo, Etty Chirolde, Laureano Peña Bazant Pérez Jose Ortega Baez, Ana Lidia Hernández Méndez; “Arnaldo Milián” Hospital, Villa Clara (101): Teresita Fleito Castex, Felicia García, Seco, Angel Alfaro, Cecilio González Benavidez; “Lucía Iñiguez” Hospital, Holguín (99): Armando González, Eneida Caraballosa, Esther Peña, Isel Sánchez Mirna Enel Pérez Muñoz; “Carlos M. de Céspedes” Hospital, Granma (95): Francisco Vázquez, José Ortega, Juan Planas Brooks, Juan Carrazona, Eberto Caravana; “Antonio Luaces Iraola” Hospital, Ciego de Ávila (87): José Hernández Cañete, Mislene Álvarez Hernández, Xiomara Herrera, Jorge Herrera Zamora; “Manuel Ascunce” Hospital, Camagüey (84): Ariel Hernández Varela, Yanor Agüero, Fidel Rivero Fernández, Raúl Romay Boitrago, Jorge Luis Valdez, Nicolás Socarras, Pedro Vejerano, Ramón Bentrogo, Misleidis González Cedeño, Nelina Morales, Odalis Escalante; “José R. López Tabranes” Hospital, Matanzas (77): Edel Fleitas Pérez, Leydis Hernández Rodríguez, Jaqueline Ramos Serpa, Martha Jiménez, Arístides García Herrera, Elizabeth Cao, Isis de la Caridad Abreu Jiménez, Daymar Bom Pérez, Yadira García; “Agostinho Neto” Hospital, Guantánamo (72): Zulema Mena, Georgina Grave de Peralta, Vladimir Sarrión; “Ernesto Guevara” Hospital, Las Tunas (58): Dianelis González, Zulema Elliot Pérez, José Luis Solis, José Luis Rivero Miranda, José Pablo Ponce; “Ambrosio Grillo” Hospital, Santiago de Cuba (57): Emilio Goulet, Lisandra Betancourt, Alina Echevarría, Jorge Lockhant; “Enrique Cabrera” Hospital, Havana (44): Heriberto Artaza Sans, Sandra González Pelegrí, Angela Blanco Díaz, Natalia Poll Marrón, Evaristo Vargas Machirán, Eduardo Atencio Soriol, Pedro Goicochea Díaz; “Julio Arestegui” Hospital, Matanzas (37): Leitter Pérez, Juan Díaz; “Julio Trigo” Hospital, Havana (36): Reinaldo Martínez Garrido; “Saturnino Lora” Hospital, Santiago de Cuba (36): Natacha Sancho Sueto, Arelis Frómeta, David Díaz, Hever Viguera, Mayelin Sabourit; “Amalia Simoni” Hospital, Camagüey (35): Alberto Álvarez Varona, Jorge Gómez , Gustavo Pérez Hechevarría, Denny González, Victor Alfonso; “Guillermo Domínguez” Hospital, Las Tunas (29): Wilber Velázquez Chacón; “Calixto García” Hospital, Havana (26): Osmel Castillo, Aimeé Rodríguez Hernández, María Campos, Remberto García, Joaquín David Liziaga Vázquez, Julio César Nuñez Vázquez; “Joaquín Castillo” Hospital, Santiago de Cuba (26): Bencay Joa Liranza; Octavio de la Concepción Hospital, Guantánamo (26): Arturo Pons; “Celia Sánchez” Hospital, Granma (24): Odelaisys Hernández Saborit, Sergio Fernández; “Victoria de Girón” Polyclinic, Santiago de Cuba (23): Carlos Calderón, Orlando San Pedro; “Carlos Font” Hospital, Holguín (22): Tamara Pérez; “Roberto Rodríguez” Hospital, Ciego de Ávila (22): Elier del Castillo, Jorge Morales Florat; “Alcides Pino” Polyclinic, Holguín (20): María Antonia Rodríguez, Tania García; “Mártires de Mayarí” Hospital, Holguín (19): Isabel Carrasco, Eneida Caraballosa; Military Hospital, Holguín (18): Pedro Matos García, Olides Cobas Díaz, Daniel Rodríguez Curí; “Diez de Octubre” Hospital, Havana (17): Deysi Acosta Lapera, Héctor Chivas, Manuel Hernández Rivero; National Institute for Angiology and Vascular Surgery, Havana (17): José Fernández Montequín , Calixto Valdez Pérez, José Llanes Barrios, Williams Savingne Gutierrez, Neobalis Franco Pérez, Daniel Reynaldo Concepción; “Comandante Manuel Fajardo” Accommodation, Havana (13): Reyna Lourdes Morejón Vega; “Manuel Fajardo” Hospital, Havana (12): Milagros Romero Gamboa, Máximo Sander López; “Orlando Pantoja” Hospital, Santiago de Cuba (12): Raúl Mesa; “Máximo Gómez” Polyclinic, Granma (12): Rafaela Rondón; “Ciro Redondo” Hospital, Artemisa (11): Odelaysis Hernández Saborí; “Aleida Fernández” Hospital, Mayabeque (11): Pedro González, Vicente Vega Mederos; “Comandante Pinares” Hospital, Artemisa (8): José Ortega Baez; “Martha Abreu” Polyclinic, Villa Clara (8): Juan Miguel García Velázquez; “XXX Anniversary” Polyclinic, Villa Clara (7): Juan Miguel García Velázquez; “Edor Reyes” Polyclinic, Granma (6): Salvador Oliva; “Roberto Fleites” Polyclinic, Villa Clara (6): Juan Miguel García Velázquez; “Miguel Enriquez” Hospital, Havana (5): Luis Olivera Baez, Justa Peñalver; “Santa Cruz del Sur” Hospital, Camagüey (5): Luis Gustavo Cisneros; Banes Hospital, Holguín (4): Antonio Ricardo García; “XXX Aniversario” Hospital, Villa Clara (4): Juan Miguel García Velázquez; Camilo Cienfuegos Polyclinic, Havana (3): Carmen Luisa Ramos; “Manuel A Varona” Polyclinic, Camagüey (3): Esteban Lopez; “Tamara Bunke” Polyclinic, Holguín (3): Luis Aguilar; “Gustavo Aldereguía” Hospital, Holguín (2): Manuel Balán; “Juan Bruno Zayas” Hospital, Santiago de Cuba (2): Rolando Castillo; “San Luis” Polyclinic, Pinar del Río (2): Aida Rosa Hernández; “XX Anniversary” Polyclinic, Villa Clara (2): Juan Miguel García Velázquez; “19 de Abril” Polyclinic, Havana (2): Reyna Lourdes Morejón Vega; “Wajay” Polyclinic, Havana (2): Angela Blanco Díaz; “Colón” Hospital, Matanzas (1): Alejandro Piedra-Fita Tejeda; “Mario Muñoz” Hospital, Matanzas (1): Maylin Torres; “Ramón Pando Ferrer” Polyclinic, Villa Clara (1): Juan Miguel García Velázquez; “Santa Clara” Polyclinic, Villa Clara (1): Juan Miguel García Velázquez; “Isabel Rubio” Polyclinic, Pinar del Río (1): Aida Rosa Hernández; “Raúl Sanchez” Polyclinic, Pinar del Río (1). Aida Rosa Hernández.

Monitoring, data management, and analyses.

Center for the Development of Pharmacoepidemiology: Isis Belkis Yera Alós, Liuba Carbonell Alonso, Julián Pérez Peña, Francisco Debesa García, Alina Alvarez Crespo, Ismary Alfonso Orta, Giset Jiménez López, Jenny Ávila Pérez, Ana García Milián, Aleida Díaz Hernández; National Pharmacoepidemiology Network: Yumara Díaz Castro (Pinar del Río), Gustavo Rodríguez (Artemisa–Mayabeque), Deborah Rodríguez Piñeiro (Havana), Adis Martín (Havana), Lia Mónica Bravo (Havana), Loida Báez (Havana), Armando Morri (Havana), Leydis Santos Muñoz (Matanzas), Miraida Baute (Cienfuegos), Luis Orlando Rico Martell (Ciego de Ávila), Orlando René Águila González (Villa Clara), Betania Rodríguez (Sancti Spíritus), Natacha Caballero (Camagüey), Soraida García (Las Tunas), Maria Elena Fernández Tablada (Holguín), Gloria Zaldivar (Holguín), Zayda Herrera López (Holguín), Yeraldis Ramírez Calzadilla (Granma), Sheila Tamayo (Santiago de Cuba), Yudeisi Trabanca Beltrán (Guantánamo); Center for Genetic Engineering and Biotechnology: Ernesto López Mola, Angela Tuero Iglesias, Carmen Valenzuela Silva, Jorge Berlanga Acosta, Miriela Gil Mena, Ricardo Silva Rodríguez, Marianela García Siverio, Francisco Hernández Bernal, Elizeth García Iglesias, Leovaldo Álvarez Falcón, María Dolores Castro Santana, Rafael Ibargollín Ulloa, Luis Herrera Martínez, Pedro Antonio López Saura.

## Abbreviations

ABI: Ankle/brachial pressure index; AE: Adverse event; CDF: Center for the development of pharmacoepidemiology; CECMED: State center for the control of medicaments, diagnostics and medical devices; CRF: Case report form; DFU: Diabetic foot ulcer; NCR: National cancer registry; rhEGF: Recombinant human epidermal growth factor; SAE: Serious adverse event; Tpw: Times per week.

## Competing interests

Authors IBYA, CMVS, ADTI, ELM, and PALS. are employees of the Center for Genetic Engineering and Biotechnology (CIGB), where rhEGF is produced and sponsored the clinical trials and postmarketing study where the data were taken from. The postmarketing study was also financed by the Ministry of Public Health of Cuba. IBYA had no conflict of interest when the study was done. The rest of the authors have no conflict of interests.

## Authors’ contributions

IBYA wrote the manuscript and participated in the protocol design, interpretation of the results, and final report writing; LAC took part in protocol design, data collection, and monitoring; CMVS made all the statistical analyses of this work, the interpretation of the results, and final report writing; ADTI participated in the statistical analyses; MMM and IMR were the site principal investigators that included more subjects and were part of the work steering committee; ELM took part in study design and organization; PALS collaborated in this manuscript writing and reviewing and contributed in protocol designs, analyses and interpretation of the results, and final report writing.

## Pre-publication history

The pre-publication history for this paper can be accessed here:

http://www.biomedcentral.com/2050-6511/14/44/prepub
